# On the Medical Properties of Nitrate of Soda

**Published:** 1854-02

**Authors:** J. B. Brown


*On the Medial properties of Nitrate of Soda.* By J. B. Brown, M. D.

This article which in many works on Materia Medica is not even mentioned, seems to be possessed of qualities too valuable to be entirely passed over, or as is done in some books, merely quoted for the purpose of calling its name. As I have made use of this drug with much success in my own practice during the last two years in this country, I deem it a duty to communicate the results to our profession.

The first notice of the physiological action of this salt I found in a medical journal for 1843, in which Dr. Zimmermann published the results of his experiments, it is as follows:

"Nitrate of Soda dissolves the protein element of the blood much less than Nitrate of Potassa, coagulated fibrin being but very little or rather not at all influenced by it, while at the same time it contracts the blood corpuscles much more firmly even to shriveling, and renders the serum redder and richer in hæmatin."

Rademacher, a Physician of Vienna, was probably the first who used this article to any very great extent as a therapeutical agent. As he attributes to it the most extraordinary properties in different diseases, claiming it as a universal remedy and recommending it in the most heterogeneous affections without stating any particular indications for its use, I was induced two years since to make some experiments myself for the purpose of ascertaining whether the medicine possessed the value which its friends had claimed for it. Rademacher thinks that it is more useful in gastric fevers than Nit. Potassæ, which as an active solvent of fibrine, causes a more rapid putrescence, and which in cases where the a priori zymotic tendency is evidently contra-indicated, whilst the Nit. Soda although it restrains the pseudoplastic process, does not produce any excessive evacuations.

When given as a gentle laxative this salt is easily borne, but when given in large quantities it produces fluid passages with tenesmus. Nevertheless according to my experience with this remedy in the prevailing diseases of this country such as irritation of the mucous membrane of the intestines and especially in acute or chronic dysentery, its value can scarcely be surpassed by any other agent of our materia medica. No remedy has so rapidly succeeded with me in restoring natural passages and relieving the extense suffering in the worst forms of this annual complaint, as Nitrate of Soda. Most of my cases, I had to treat only four or five days, when all the symptoms under the influence of this specific, if I may use this term, disappeared entirely. Other cases, in which a cure was not so speedily brought about, were comparatively rare, and I found this fact generally owing to a fault of diet or some other unfavourable circumstance of the patient. Nevertheless I have been so fortunate, as never to treat any case of dysentery longer than at most for a fortnight, whilst out of 6 cases I generally restored 5 of them to health, within the above mentioned limited space of a few days. It is not my purpose to enter into a detail of the treatment of this disease, but I will confine myself here to a single remark, that of all the remedies in use against it, there is no one existing, to my knowledge, safer, quicker and more innocent in its agency, than the one under consideration.

The formula I usully employ is this :

| | | |
|---|---|---|
| ℞ | Ol. Amygdal. dulc. | ʒiv to vj |
| | Gum. Mimos. | ʒij to iij |
| | Aq. distillat. | ℥jv to vj |
| | Fiat lege artis emulsio, cui adde | |
| | Sodæ Nitratis | aa |
| | Aquæ lauro-Cerasi | ℥ss |
| | Syr. Simp. | ℥j |

M. S.—A table-spoonful to be taken every one to two hours, after having been well shaken.

For chidren I prescribe the half or only third part of the quantity, and I found it an excellent means for overcoming in their different forms diarrhœa and dysentery, at least for the purpose of diminishing the irritability of the bowels, after which other remedies, according to the indications of the case, might be made use of.

After the evacuations have become normal, the medicine is to be continued for one or two days, on account of a great liability to relapse, which not unfrequently may very disagreeably surprise both the patient and physician. But this disposition is owing to the disease itself, as every practitioner will agree, and not to the specific effect of Nitrate of Soda. In some cases of the most severe characters I ordered the addition of a few grains of the Extract of Opium to the former mixture, in order to allay more rapidly the extremely painful tenesmus and the nervous excitement in general. I prefer the extract to the opium in substance, because it has a milder effect, quieting the system more and not producing that peculiar venous orgasmus, which opium itself does. But I must remark here that even this milder preparation of opium ought never to be used for several days in succession for reasons I can not now explain, being beyond the limits of my present purpose. One of my patients, who suffered in a dreadful manner, after taking a mixture of brandy with pepper, in order to stop the whole matter at once, as he thought, had to take the above described portion four times one after the other; within 7 days he was perfectly restored.

I have been now in more than a hundred cases of dysentery so successful, as to restore my patients within the period of from 4 to 14 days at most, under the principal influence of Nitrate of Soda, never using a single grain of calomel, which I avoid for fear of salivation and of its consequence, these being often as bad a complaint as the former, or even still worse, I would therefore recommend this salt as a very powerful and innocent substitute for calomel in all the different forms of dysenteric or congestive diarrhœa and dysentery.

I would mention, en passant, that tonics or astringents are to be employed, whenever, after the disappearance of the characteristic mucous discharges, a tendency to a diarrhœa from atony manifests isself, either spontaneously or in consequence of an improper diet.

